# Evidences of Basal Lactate Production in the Main White Adipose Tissue Sites of Rats. Effects of Sex and a Cafeteria Diet

**DOI:** 10.1371/journal.pone.0119572

**Published:** 2015-03-05

**Authors:** Sofía Arriarán, Silvia Agnelli, David Sabater, Xavier Remesar, José Antonio Fernández-López, Marià Alemany

**Affiliations:** 1 Department of Nutrition and Food Science, Faculty of Biology, University of Barcelona, Barcelona, Spain; 2 Institute of Biomedicine of the University of Barcelona, Barcelona, Spain; 3 CIBER Obesity and Nutrition, Barcelona, Spain; East Tennessee State University, UNITED STATES

## Abstract

Female and male adult Wistar rats were fed standard chow or a simplified cafeteria diet for one month. Then, the rats were killed and the white adipose tissue (WAT) in four sites: perigonadal, retroperitoneal, mesenteric and subcutaneous (inguinal) were sampled and frozen. The complete WAT weight in each site was measured. Gene expression analysis of key lipid and glucose metabolism enzymes were analyzed, as well as tissue and plasma lactate and the activity of lactate dehydrogenase. Lactate gradients between WAT and plasma were estimated. The influence of sex and diet (and indirectly WAT mass) on lactate levels and their relationships with lactate dehydrogenase activity and gene expressions were also measured. A main conclusion is the high production of lactate by WAT, practically irrespective of site, diet or sex. Lactate production is a direct correlate of lactate dehydrogenase activity in the tissue. Furthermore, lactate dehydrogenase activity is again directly correlated with the expression of the genes *Ldha* and *Ldhb* for this enzyme. In sum, the ability to produce lactate by WAT is not directly dependent of WAT metabolic state. We postulate that, in WAT, a main function of the lactate dehydrogenase path may be that of converting excess available glucose to 3C fragments, as a way to limit tissue self-utilization as substrate, to help control glycaemia and/or providing short chain substrates for use as energy source elsewhere. More information must be gathered before a conclusive role of WAT in the control of glycaemia, and the full existence of a renewed glucose-lactate-fatty acid cycle is definitely established.

## Introduction

Lactate is the main by-product of peripheral organ utilization of glucose under conditions of anaerobiosis / hypoxia [1] or when used by glycolytic obligatory cells such as mammalian erythrocytes [2]. Lactate is also an acidifying factor modulating oxygen release in hypoxic tissues (Bohr effect) [3]. Lactate is one of the main splanchnic (essentially liver) organs' substrate for gluconeogenesis [4], and helps transfer "oxygen debt" from glycolytic muscle during exercise to the liver, completing a Cori cycle [5]. Lactate is, also, a good substrate for lipogenesis in the liver [6] and other tissues [7,8].

Lactate is a main by-product of most tumour cells, which show high rates of glycolysis [9] due to the Warburg effect [10]. In addition, lactate may be used as energy substrate by a number of tissues: heart [11], brown and white [12] adipose tissues, muscle [13], glia [14] and others, including the biota [15], because of its rapid conversion to pyruvate. Lactate is the main circulating 3C substrate, and cell (cytoplasmic) reducing power exporter, reflecting NADH status, and acting as a marker and modulator of oxygen availability and utilization [16]. These multiple roles place lactate at the centre of a critical metabolic switch. The changes in the rate of synthesis, transport in the blood and final disposal deeply modulate (and/or are the consequence of) important metabolic substrate shifts.

The main form of adipose tissue, white adipose tissue (WAT) is usually associated with energy storage in the form of an enormous vacuole containing essentially triacylglycerols. However, a significant part of body fat reserves are not found in the main WAT sites, but present in other cell types, such as myocytes or hepatocytes [17] or, more commonly, as WAT interspersed between other cell types in a number of tissues [18–21]. Curiously, all body reserves' mass changes are correlated between the macroscopic and disperse triacylglycerol depots, which, this way, show a remarkable uniformity in its physiological function as reserve organ [22], irrespective of variation in additional specialised functions [23,24].

We have recently observed that cultured 3T3L1 adipocytes, in the absence of undifferentiated fibroblasts, use glucose at extremely high rates, releasing lactate under conditions of full normoxia [25]. This is in agreement with *in vivo* lactate production by WAT [26–28] parallel to its low consumption of oxygen [29,30]. The limiting factor for lactate production under high medium glucose seems to be the availability of ADP [25]. The WAT breakup of 6C glucose to two 3C units may be directed to decrease glucose availability [25] in order to help protect the tissue from excess substrate and the damaging hypertrophy caused by excess energy substrates [31]. This defence mechanism may help restrict the supply of substrates (mainly glucose), but also that of oxygen, possibly inducing hypoxia, which has been suggested to favour the development of inflammation [32,33] and the consequent WAT immune response [34]. However, the limited needs of oxygen of adipocytes [25], and the low WAT oxygen consumption [30] suggest that the development of inflammation in WAT may not be directly related to oxygen supply.

In the present study we intended to find whether WAT lactate production *in vivo* may be in some way related to the excess energy supply of high-energy diets, but also to check whether sex [35] may also affect the WAT breakup of glucose to 3C units.

## Materials and Methods

### Animals, diet and experimental setup

All animal handling procedures and the experimental setup were in accordance with the animal handling guidelines of the corresponding European and Catalan Authorities. The Committee on Animal Experimentation of the University of Barcelona authorized the specific procedures used in the present study.

Nine week old female and male Wistar rats (Harlan Laboratory Models, Sant Feliu de Codines, Spain) were used. Six animals per group were housed in cages (two same-sex rats in each) with wood shards for bedding. They had free access to water and were kept in a controlled environment (lights on from 08:00 to 20:00; 21.5–22.5°C; 50–60% humidity). Two groups of animals for each sex were selected randomly, and were fed *ad libitum*, for 30 days, with either normal rat chow (type 2014, Harlan) or a simplified cafeteria diet as previously described [36].

The experimental setup consisted, thus, of four groups of 6 rats each: male-control, female-control, male-cafeteria and female-cafeteria. Rat weight and food consumption were measured, and we found that these parameters followed closely previous studies done using the same experimental setup [22]. Diet composition was (expressed as energy content): carbohydrate 67%, protein 20%, and lipid 13% for controls; the mean composition of the cafeteria diet ingested was: carbohydrate 47%, protein 12% and lipid 41%.

In a complementary study to analyse tissue blood flows, we used two additional groups of male rats, from the same supplier, stock, and age; they were subjected to the same feeding protocols (control or cafeteria diet) for 30 days. These animals were used exclusively for the measurement of tissue blood flow as described below.

### Tissue sampling

At the end of the experiment, on day 30, the animals were anesthetized with isoflurane at the beginning of a light cycle, and blood was drawn from the exposed aorta using dry heparinized syringes, killing the animals by exsanguination. Blood was centrifuged 20 min at 2000x*g*, at 2–4°C. Plasma was frozen and kept at -20°C.

The rats were rapidly dissected, taking large samples of mesenteric (ME), perigonadal (periovaric in females and epididymal in males, PG), retroperitoneal (RP) and subcutaneous (inguinal fat pads, SC) WAT, which were frozen in liquid nitrogen. The samples were weighed, and stored at -80°C until processed. Later, the dissection of the dead rats continued, carefully extracting the remaining WAT in ME, PG and RP sites; the rats were skinned, and the subcutaneous WAT was dissected completely. The weights of WAT thus dissected were added to those of the frozen samples to know the mass of the four WAT sites.

### WAT cellularity

Portions of frozen WAT were used for the estimation of total DNA with a classical chemical method [37]. Since a rat cell contains 5.6 pg of DNA [38], we were able to calculate the approximate number of cells per unit of WAT volume. We knew the weights and the density: ~0.9 g/mL [39] of WAT, and thus we determined the number of cells per g of tissue, and in the whole site, as well as their mean size, simply dividing the tissue volume by the number of cells.

Tissue protein content was estimated with a reliable biuret-Folin method [40]. After development of colour, turbidity was eliminated by adding to the tubes small amounts of finely powdered solid MgO, which adsorbed the suspended fat remnants, and centrifuging the tubes before reading their absorbance.

### Estimation of tissue lactate

Samples of WAT tissue (in the range of 50 mg) were homogenized, still frozen, with a tissue disruptor (IKA-T10 basic Ultra-Turrax, IKA, Stauffen Germany) in 1 ml of chilled acetone:water mixture, to a final proportion (including the expected water content of the samples) of 1.25:1 [41]. The homogenate was centrifuged; all proteins removed with the precipitate, and floating lipids, not soluble in the diluted acetone, were discarded. The acetone tissue extract and plasma (10μL samples) were used for the estimation of lactate (kit 1001330 Spinreact, Sant Esteve d'en Bas, Spain), using sodium L-lactate (Sigma-Aldrich) as standard.

The proportion of lipid per g of tissue in rats treated with the same experimental setup is known [22]. By discounting the mass of fat and protein from that of tissue we were able to obtain an estimate of the amount of water present in the WAT samples (in the range of 20%). The moles of lactate per mL of tissue or plasma and the amount of water in that same volume allowed us to calculate the molal concentrations of lactate in plasma and tissues, and to establish a molal concentrations ratio for each individual rat WAT site.

### Measurement of lactate dehydrogenase activity

A modified UV method was used [42]. Frozen tissue samples were homogenized, using the tissue disruptor, in 10 volumes of chilled Krebs-Ringer bicarbonate solution, pH 7.8 containing 5 mM dithiothreitol, 0.5% bovine serum albumin, 1% dextran (MW 200,000), 0.1% Triton X-100, and 1 mM EDTA. Freshly (i.e. not later than 3 h after thawing/homogenisation in the buffer) prepared homogenates were used for all enzyme activity measurement incubations, carried out at 25°C. Homogenates were centrifuged at 4°C and 5,000x*g* for 10 minutes to obtain a clear intermediate phase (precipitated debris and floating lipid were discarded). The reaction mixture contained 150 μM NADH, 125 mg/L bovine serum albumin and 1 mM sodium pyruvate (all products were obtained from Sigma-Aldrich). The proportion of original tissue in each measuring well was in the range of 2.0–2.5 mg, in a volume of 0.02 mL of homogenate, diluted to the adequate proportions with homogenization medium. Absorbance at 340 nm was measured at intervals of 30 s for up to 10 min. In each case, the decrease in absorbance due to the formation of NAD^+^ was plotted, and initial (V_0_) activities were determined from the course of the reaction at different times. V_0_ was assumed to correspond to V_max_ under the described conditions of analysis. Protein content was measured in each of the homogenates used for enzyme activity estimation, and used for the presentation of enzyme activities, expressed in nkat/g of protein to allow comparisons between samples with different fat content.

### Gene expression analysis

Total tissue RNA was extracted from the frozen tissue samples using the Tripure reagent (Roche Applied Science, Indianapolis IN USA), and were quantified in a ND-100 spectrophotometer (Nanodrop Technologies, Wilmington DE USA). RNA samples were reverse transcribed using the MMLV reverse transcriptase (Promega, Madison, WI USA) system and oligo-dT primers.

Real-time PCR (RT-PCR) amplification was carried out using 10 μL amplification mixtures containing Power SYBR Green PCR Master Mix (Applied Biosystems, Foster City, CA USA), 4 ng of reverse-transcribed RNA and 150 nM of primers. Reactions were run on an ABI PRISM 7900 HT detection system (Applied Biosystems) using a fluorescent threshold manually set to 0.15 for all runs.

A semi-quantitative approach for the estimation of the concentration of specific gene mRNAs per unit of tissue weight was used [43]. *Rplp0* was the charge control gene [44,45]. We expressed the data as the number of transcript copies per gram of protein in order to obtain comparable data between the groups. The genes analysed and a list of primers used is presented in Table 1.

**Table 1:** Primers used for the analysis of gene expression in WAT of control and cafeteria diet-fed rats.

gene	Protein	5' > 3'	3' > 5'	bp
*Ldha*	lactic acid dehydrogenase (muscle type)	CACTGGGTTTGAGACGATGA	GTCAGCAAGAGGGAGAGAGC	125
*Ldhb*	lactic acid dehydrogenase (heart type)	CCAGGAACTGAACCCAGAGA	TCATAGGCACTGTCCACCAC	131
*Glut4*	glucose transporter 4	CTTGATGACGGTGGCTCTGC	CACAATGAACCAGGGGATGG	127
*Hk2*	hexokinase 3	ATTCACCACGGCAACCACAT	GGACAAAGGGATTCAAGGCATC	113
*G6pd*	glucose-6P dehydrogenase	GACTGTGGGCAAGCTCCTCAA	GCTAGTGTGGCTATGGGCAGGT	77
*Pdk4*	pyruvate dehydrogenase kinase type 4	GTCAGGCTATGGGACAGATGC	TTGGGATACACCAGTCATCAGC	137
*Pdk2*	pyruvate dehydrogenase kinase type 2	TCACTCTCCCTCCCATCAA	CGCCTCGGTCACTCATTT	75
*Nos3*	nitric oxide synthase (endothelial type)	CAAGTCCTCACCGCCTTTT	GACATCACCGCAGACAAACA	138
*Acc1*	acetyl-CoA carboxylase type 1	AGGAAGATGGTGTCCGCTCTG	GGGGAGATGTGCTGGGTCAT	145
*Fas*	fatty acid synthase	CTTGGGTGCCGATTACAACC	GCCCTCCCGTACACTCACTC	163
*Acly*	ATP: citrate lyase	GACCAGAAGGGCGTGACCAT	GTTGTCCAGCATCCCACCAGT	96
*Hsl*	hormone-sensitive lipase	CCCATAAGACCCCATTGCCTG	CTGCCTCAGACACACTCCTG	94
*Lpl*	lipoprotein lipase	GAAGGGGCTTGGAGATGTGG	TGCCTTGCTGGGGTTTTCTT	103
*Atgl*	triacylglycerol lipase (adipose tissue)	CGGTGGATGAAGGAGCAGACA	TGGCACAGACGGCAGAGACT	138
*Cpt1*	carnitine palmitoleoyl transferase (liver)	CCGCTCATGGTCAACAGCA	CAGCAGTATGGCGTGGATGG	105
*Cpt2*	carnitine palmitoleoyl transferase (muscle)	TGCTTGACGGATGTGGTTCC	GTGCTGGAGGTGGCTTTGGT	152
*Lcad*	long-chain acyl-CoA dehydrogenase	ATGCCAAAAGGTCTGGGAGT	TCGACCAAAAAGAGGCTAATG	148
*Rplp0*	60S acidic ribosomal protein 0	GAGCCAGCGAAGCCACACT	GATCAGCCCGAAGGAGAAGG	62

### Measurement of tissue blood flows

On day 27, the rats were implanted with two cannulas through the left carotid artery using Intramedic PE-10 polyethylene tubing (Becton Dickinson, Parsipanny, NJ USA), under isoflurane anaesthesia. The first carotid cannula was used to draw blood from descending aorta and the other to inject microspheres directly into the heart outflow following the instructions provided by the supplier. At 12 h intervals, the viability of the cannulas was checked (without disturbing the animals) by drawing blood up a few mm, followed by refilling with heparinized saline. On day 30 the rats were transferred to smaller individual cages, shielded from the operators. Two hours later, they were injected through the left ventricle cannula with 10^5^ red latex beads (Molecular Probes, Carlsbad, CA USA) suspended in 0.1 ml of 9 g/L NaCl. At the same time, blood (about 0.2 mL) was slowly drawn (exactly during 60 s) through the other cannula. The rats were, then, anesthetized with isoflurane, and larger blood samples were obtained from the exposed aorta. Blood and tissue samples were frozen and kept at -80°C. After sacrifice the position of the cannulas was checked; no placement errors, nor cannula clotting were found. The weights of all organs analysed were measured.

Blood and tissue samples of known volume/weight were digested with 4 M KOH for 24 hours at 25°C with occasional stirring. The samples were filtered through glass-fibre filters (GF/D, 2.5 μm, Whatman, Maidstone, Kent UK) and rinsed with Tween-20 (20 g/L) followed by distilled water. Then, the fluorospheres were extracted from the filter with 2.5 ml of etoxyethyl acetate. The fluorescence at red (565 nm) excitation wavelength was measured at 598 nm emission wavelength in a spectrofluorimeter. Samples were adequately diluted and compared against tissue blanks, made with pieces of the same tissues from rats of the first experiment, which had not received fluorescent beads. At least two samples and/or extractions for each tissue were used / carried out. Samples were processed according to the bead supplier specifications. The number of beads in tissue samples was estimated from the organic extract fluorescence. The distribution of bead fluorescence equivalents vs. bead controls was used to obtain a percent distribution of blood flow between organs, since the amount of beads injected was known: a sample of the injected material was also analysed to correct for possible errors in the evaluation of injected bead numbers.

Calculation of cardiac output was done by measuring the amount of beads in the blood drawn for one minute from the artery. Bead concentration and the known amount of beads injected allowed the calculation of cardiac output. Absolute blood flows were calculated from the number of beads leaving the heart per unit of time and blood volume and the percentage of beads retained in the organs of the rats.

### Statistics

Comparisons between groups were done with two- or three-way ANOVA analyses using the Statgraphics Centurion XVI software (Warrenton, VA USA). Analyses of correlations and curve fitting (i.e. for V_0_ estimation) were carried out using Prism 5 program (GraphPad Software, San Diego CA USA).

## Results

The initial and final weights, energy intake, and the basic plasma parameters are shown in Table 2. Body weight changes and energy data just repeat what we have previously published [22]. Plasma glucose, triacylglycerols, total cholesterol and urea were within the normal range, showing no significant differences between groups, except for higher glucose and lower urea in cafeteria diet-fed rats.

**Table 2:** Body weight, energy intake and plasma metabolites of control and cafeteria diet-fed rats.

tissue	units	male control	male cafeteria	female control	female cafeteria	P sex	i	P diet
Initial body weight	g	241±6	256±6	161±6	173±7	<0.0001		NS
Final body weight	g	372±6	420±20	232±8	277±15	<0.0001		<0.0001
Energy intake	MJ/30d	8.71±0.45	19.7±0.99	6.33±0.39	17.9±0.98	<0.0001		0.0121
Plasma glucose	mM	7.80±0.32	8.25±0.33	6.60±0.26	8.78±0.24	NS	i	0.0002
Plasma triacylglycerols	mM	1.50±0.06	1.50±0.01	1.69±0.06	1.51±0.03	0.0390		NS
Plasma cholesterol	mM	1.97±0.07	2.28±0.21	1.98±0.16	2.07±0.19	NS		NS
Plasma urea	mM	3.90±0.17	3.82±0.20	5.13±0.25	3.78±0.20	0.0094	i	0.0025

The data are the mean ± sem of 6 different animals. Up to three significant digits are shown for each mean value. Statistical significance of the differences between groups (2-way anova): the columns show the P values for sex, diet and their interaction; a i in the interaction column indicates a significant interaction between the two factors analysed.

Table 3 shows the WAT site mass cellularity and protein content for female and male rats subjected to control and cafeteria diets. There were significant differences between sites for total mass, mean cell size, number of cells per unit of tissue weight and in the whole site. Sex effects were limited to SC and RP sites' mass and cell content; PG WAT also showed significant changes linked to sex for site cell and protein content. No effects attributable to sex were observed on ME WAT. With respect to the effects of diet, all sites showed significant differences for all the parameters included in Table 3, except for RP WAT protein content.

**Table 3:** White adipose tissue mass and cell size in adipose tissue sites of control and cafeteria diet-fed rats.

parameter	units	site	male control	male cafeteria	female control	female cafeteria	P-site	P sex	i	P diet
WAT site mass	g	SC	12.3±0.2	19.9±1.0	7.02±0.25	12.3±0.6	<0.0001 S>P>M≈R	<0.0001		<0.0001
		ME	4.94±0.64	8.38±0.95	3.92±0.33	9.02±1.25		NS		<0.0001
		PG	7.34±0.50	12.8±1.6	4.83±0.39	11.8±1.70		NS		<0.0001
		RP	6.29±0.80	9.98±1.38	2.79±0.35	7.81±0.77		0.0051		0.0001
mean cell size	nL	SC	7.72±0.88	8.34±0.49	7.15±0.39	9.89±0.25	<0.0001 R>P≈S>M	NS		0.0088
		ME	6.76±0.21	7.12±1.28	4.19±0.84	9.33±0.61		NS	i	0.0065
		PG	7.72±0.66	9.16±0.84	8.35±0.58	11.1±0.6		NS		0.0061
		RP	9.74±0.25	9.48±0.56	9.43±0.78	12.1±0.4		NS	i	0.0439
number of cells in the whole site	10^6^·cells	SC	1474±121	2556±69	1000±73	1473±213	<0.0001 S>P≈M>R	<0.0001	i	<0.0001
		ME	658±69	1392±265	906±129	959±116		NS		0.0411
		PG	883±25	1382±85	579±31	1069±145		0.0019		<0.0001
		RP	681±65	1072±165	298±37	639±46		0.0003		0.0009
number of cells /g tissue	10^6^·cells/g	SC	122±9	122±8	142±7	101±3	0.0005 M>S≈P≈R	NS	i	0.0122
		ME	149±5	134±21	230±35	109±7		NS	i	0.0051
		PG	134±10	104±7	123±8	87±4		NS		0.0007
		RP	103±3	102±5	110±9	83.0±2.6		NS	i	0.0262
protein content/g tissue	mg/g	SC	63.1±11.6	35.0±3.9	51.2±3.8	41.2±4.4	<0.0001 M>R>S≈P	NS		0.0105
		ME	74.2±7.4	60.9±4.7	86.2±4.2	57.5±2.9		NS		0.0010
		PG	44.3±1.6	35.0±2.0	54.4±2.4	47.6±2.6		<0.0001		0.0015
		RP	65.1±6.3	63.7±5.2	62.9±4.7	50.7±1.3		NS		NS

The data are the mean ± sem of 6 different animals. Up to three significant digits are shown for each mean value. SC/S = subcutaneous inguinal: ME/M = mesenteric; PG/P = perigonadal (periovaric, epididymal), RP/R = retroperitoneal. Statistical significance of the differences between groups (2-way anova): the columns show the P values for sex, diet and their interaction. A i in the interaction column indicates a significant interaction between the two factors analysed. The statistical significance of differences between sites was estimated by a 3-way-ANOVA. P values >0.05 are presented as NS; the sequences indicate overall significant differences between sites; post-hoc Duncan test: the sign > indicates significantly higher values at the left; commas are equivalent to NS.

Rats fed the cafeteria diet had larger WAT depots, but the relative size order was unchanged. Mean cell size followed the same pattern in all four groups, irrespective of global higher sizes in cafeteria diet-fed rats: RP>PG≈SC>ME.

**Fig 1:**
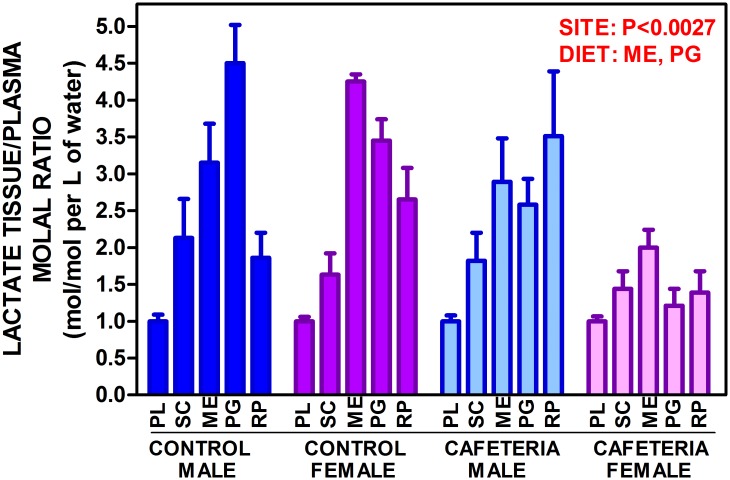
Lactate adipose tissue/plasma molal ratio of control- and cafeteria diet-fed male and female rats. The data are the mean ± sem of 6 animals per group, and are expressed in moles/L of water in the tissue divided by moles/L of water in plasma. PL = plasma; SC = subcutaneous WAT; ME = mesenteric WAT; PG = perigonadal WAT; RP = retroperitoneal WAT. Statistical differences between groups: two-way anova for sex and diet and three-way anova for "site" are marked in red.

Tissue lactate levels (Table 4) were lower in tissue (μmol/g) than in plasma (mM), and were not affected by sex, except for RP. Cafeteria diet decreased tissue (and plasma) lactate (significant for plasma, ME and PG). When the data were expressed in molal units, the picture was quite different (Fig. 1). The molal concentration ratios for WAT versus plasma were in all cases higher than 1, i.e. tissue lactate concentration in the water available (i.e. discounting protein and fat) was up to four-fold higher than in arterial plasma. There was a significant effect of site and of diet for PG and ME, with no effects of sex. The patterns of molal ratios between tissue and plasma lactate, however, were different in males PG>ME>SC>RP and females ME>PG>RP>SC. Cafeteria diet changed the patterns to RP>ME>PG>SC in males and ME>SC>RP>PG in females.

**Table 4:** Lactate levels in plasma and adipose tissue sites of control and cafeteria diet-fed rats.

tissue	units	male control	male cafeteria	female control	female cafeteria	P sex	i	P diet
plasma	mM	3.10±0.29	2.64±0.21	3.78±0.24	2.57±0.21	NS		0.0028
subcutaneous WAT	μmol/g	2.16±0.71	1.57±0.35	2.37±0.37	1.43±0.22	NS		NS
mesenteric WAT	μmol/g	1.98±0.33	1.80±0.37	2.87±0.17	1.04±0.13	NS	i	0.0019
perigonadal WAT	μmol/g	2.00±0.22	1.16±0.26	2.12±0.12	0.45±0.10	NS		<0.0001
retroperitoneal WAT	μmol/g	0.803±0.152	0.781±0.134	1.58±0.21	0.522±0.116	0.0026	i	NS

The data are the mean ± sem of 6 different animals. Up to three significant digits are shown for each mean value. Statistical significance of the differences between groups (2-way anova): the columns show the P values for sex, diet and their interaction; a i in the interaction column indicates a significant interaction between the two factors analysed. The statistical significance of differences between sites was estimated by a 3-way-ANOVA; the P value was <0.0001; post-hoc Duncan test (same conventions as in Table 3): plasma > mesenteric, subcutaneous > perigonadal > retroperitoneal).

**Fig 2:**
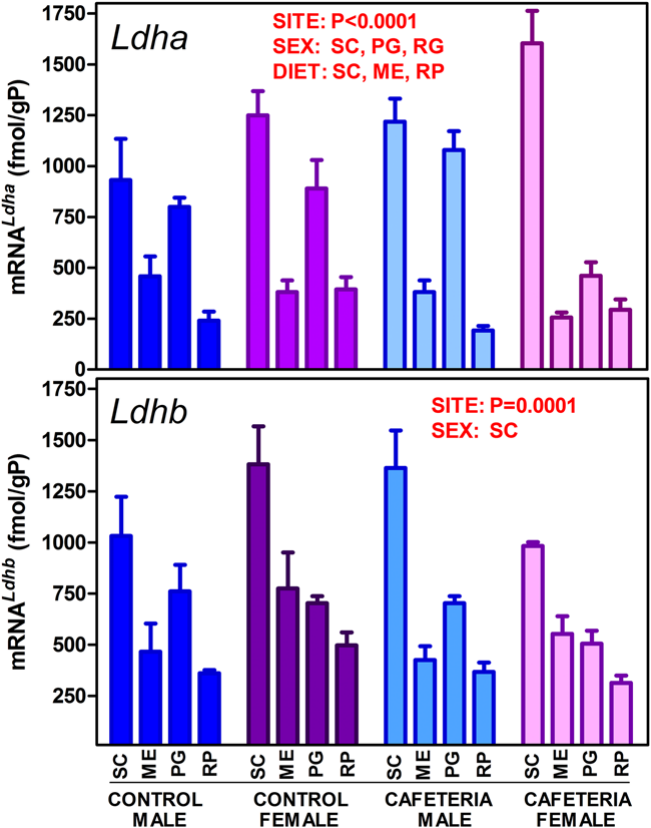
Lactate dehydrogenase genes (*Ldha* and *Ldhb*) expression in WAT sites of female and male rats fed control or cafeteria diet. The data are the mean ± sem of 6 animals per group, and are expressed in fmol/gP (g of tissue protein) to render the figures comparable. The conventions used are the same as in Fig. 1.

**Fig 3:**
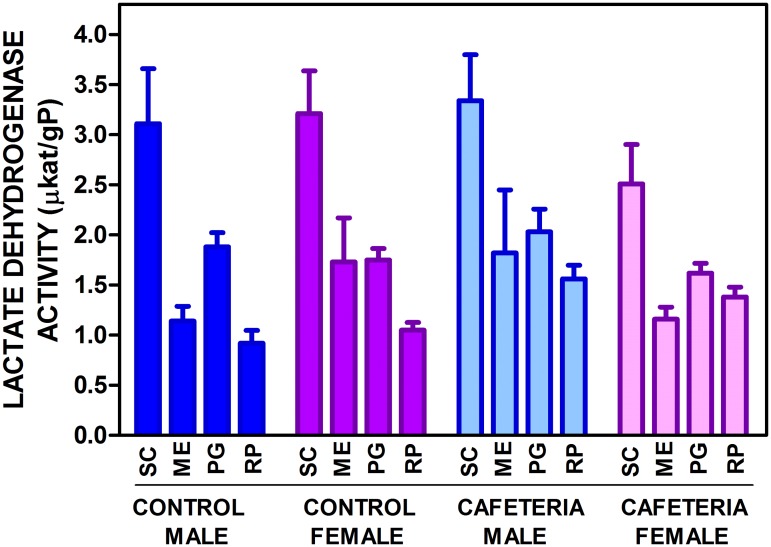
Total tissue lactate dehydrogenase activity in WAT sites of female and male rats fed control or cafeteria diet. The data are the mean ± sem of 6 animals per group, and are expressed in μKat/gP (g of tissue protein) to render the figures comparable. The conventions used are the same as in Fig. 1.

The levels of expression of the genes for the main lactate dehydrogenase isoenzymes in WAT (*Ldha* and *Ldhb*) are presented in Fig. 2. There was a marked difference in expression of both genes depending on WAT site. The patterns of distribution were fairly similar for both genes, with maximal expression in SC, and minimal in RP. However, there were significant differences induced by sex and diet. The effect of sex was less marked on *Ldhb*, which did not show significant effects of diet either, in contrast with *Ldha*. The actual tissue enzyme activities are shown in Fig. 3. The patterns were comparable to those of *Ldha* and *Ldhb* expressions, but we must assume that the lactate dehydrogenase activity measured in tissue (in fact V_max_ under the conditions of measurement) was the result of the sum of both isoenzyme activities. No differences were observed for sex and only RP showed a significant effect of diet; however, the variable "site" was highly significant as in the gene expression analysis. In all groups, the patterns were comparable, but not identical, with maximal enzyme activity in SC.

**Fig 4:**
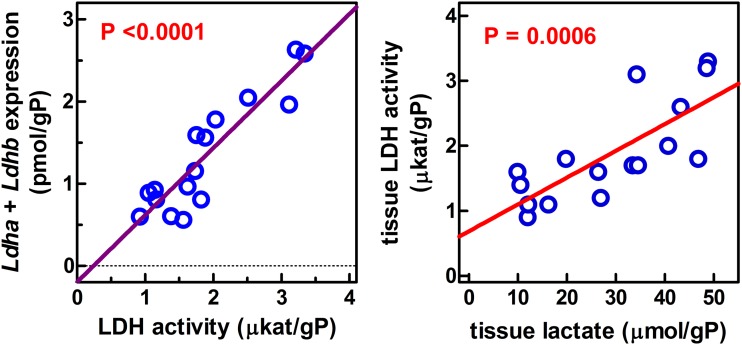
Linear correlation analysis of the relationship between lactate dehydrogenase activity and expression or tissue lactate content in WAT sites of male and female rats fed a control or cafeteria diet. LDH = lactate dehydrogenase; gP = g of protein. Each circle represents the mean value for one group of rats (i.e. female/male, control/cafeteria). Enzyme activities are those presented in Fig. 3; expression data are the sum of *Ldha* and *Ldhb* (Fig. 2) for the corresponding groups; tissue lactate were those shown in Table 4. Correlation between lactate dehydrogenase activity and gene expression: r^2^ = 0.828, significantly different from zero (P<0.0001). When all the individual data (i.e. all sites and groups) were plotted, the corresponding values were r^2^ = 0.380, and P<0.0001. The separate analyses of *Ldha* and *Ldhb* (using the mean values) gave similar results *Ldha* r^2^ = 0.642, P = 0.0002, and *Ldhb* r^2^ = 0.840, P<0.0001. Correlation between tissue lactate dehydrogenase activity and lactate content: r^2^ = 0.583, significantly different from zero (P = 0.0006). When all the individual data (i.e. all sites and groups) were plotted instead of only the means, the corresponding values were r^2^ = 0.307, and P<0.0001.

The comparison of enzyme activity vs. the expression of the genes for the two isoenzymes implicated in that activity could not be done in a direct way, since gene expression of an enzyme seldom can be directly correlated with its activity in a tissue. However, we plotted (Fig. 4) the mean values for lactate dehydrogenase enzyme activity per g of tissue protein of all groups (i.e. different sex and diet) *vs*. the sum of the corresponding *Ldha* and *Ldhb* expressions, also referred to g of tissue protein. We obtained a significant correlation between expression and enzyme activity (P<0.0001 vs. zero). When the individual rat data were plotted, we obtained a little more dispersion, but the slope of the regression line and the P values vs. zero were practically unchanged. The separate analysis of *Ldha* and *Ldhb* gave similar results (the slopes were practically halved with respect to the sum of expressions, but were superimposable for both genes). In both cases, the correlation was also significant. These data point at a direct relationship between the expression of both genes and the enzyme activity, but also that their contribution to final lactate dehydrogenase activity was similar for both isoenzymes (heart type and muscle type); thus the isozyme pattern of rat WAT is HHMM. When lactate dehydrogenase activity was plotted versus tissue lactate expressing both entities per g of protein, a significant correlation was observed between both parameters.

Table 5 presents the expression data for enzymes related with glucose oxidation. As observed for the lactate dehydrogenase genes, there was a marked significant effect of site in all cases. There were no effects of sex on *Glut4*, but females had higher hexokinase 2 expression, significant in ME and RP. The control of glucose entry in the cell was more affected by diet: *Glut4* and *Hk2* expressions were significantly lower for all cafeteria diet groups with the exception of SC. Glucose-6P dehydrogenase gene (*G6pd*) expression was also lowered by cafeteria diet, significantly for ME and RP, the latter with higher values in females.

**Table 5:** Gene expression in WAT of control and cafeteria diet-fed rats in fmol/g protein. I Glucose oxidation and nitric oxide synthase.

protein	gene	WAT	male control	male cafeteria	female control	female cafeteria	P site	P sex	i	P diet
glucose transporter 4	*Glut4*	SC	108±29	123±25	161±184	115±8	<0.0001 S≈P>M≈R	NS		NS
		ME	31.2±8.6	15.8±3.0	116±36	12.2±0.6		NS		0.0189↓
		PG	109±8	70.2±2.7	160±54	32.6±6.6		NS		0.0134↓
		RP	44.0±6.5	25.2±3.9	119±43	26.0±5.8		NS		0.0088↓
hexokinase 2	*Hk2*	SC	11.7±2.5	13.0±1.9	18.9±3.7	14.7±0.9	<0.0001 S≈P>M≈R	NS		NS
		ME	7.9±1.2	2.7±0.5	16.8±3.2	1.5±0.2		0.0304^f^	i	<0.0001↓
EC 2.7.1.1		PG	16.9±2.8	14.6±2.0	25.6±5.7	10.5±2.1		NS		0.0266↓
		RP	4.1±0.4	2.9±0.4	13.5±4.5	3.3±0.6		0.0084^f^	i	0.0030↓
glucose-6P-dehydrogenase	*G6pd*	SC	334±73	413±51	257±25	302±36	0.0001 S>P>M≈R	NS		NS
		ME	129±18	75.6±9.3	202±32	62.9±4.6		NS	i	<0.0001↓
EC 1.1.1.49		PG	129±12	188±23	285±51	145±16		NS	i	NS
		RP	84.2±14.2	67.0±5.7	183±35	82.0±9.3		0.0048^f^		0.0037↓
pyruvate dehydrogenase kinase 4	*Pdk4*	SC	181±73	187±41	22.7±2.6	90.2±21.6	<0.0001 S>P≈M≈R	0.0113^m^		NS
		ME	14.2±3.7	14.4±2.1	7.6±3.9	9.1±1.7		NS		NS
EC 2.7.11.2		PG	14.5±5.4	20.6±3.2	8.5±3.2	11.8±2.2		NS		NS
		RP	13.5±3.5	12.9±1.6	6.1±1.0	17.6±3.7		NS		NS
pyruvate dehydrogenase kinase 2	*Pdk2*	SC	263±87	167±41	146±13	302±36	<0.0001 S>M≈P≈M>R	NS		NS
		ME	120±24	34.2±1.7	193±51	22.9±5.3		NS		0.0009↓
EC 2.7.11.2		PG	91.7±12.8	90.1±11.4	86.6±16.8	41.8±3.1		0.0426^m^		NS
		RP	48.6±7.6	27.9±3.9	63.9±10.4	31.5±6.1		NS		NS
nitric oxide synthase (endothelial type)	*Nos3*	SC	75.4±9.5	88.9±6.0	43.2±2.8	65.5±7.2	<0.0001 S>P>M≈R	0.0009^m^		0.0190↑
		ME	21.6±2.6	15.3±2.3	18.5±1.0	10.4±1.5		NS		0.0054↓
EC 1.14.13.39		PG	31.6±6.3	32.9±4.6	22.6±2.4	13.3±1.9		0.0030^m^		NS
		RP	14.7±1.1	11.6±1.7	16.1±2.2	9.4±1.2		NS		0.0051↓

The data are presented as fmol of the corresponding mRNA for g of tissue protein, and are the mean ± sem of 6 different animals. Up to three significant digits are shown for each mean value. SC/S = subcutaneous inguinal: ME/M = mesenteric; PG/P = perigonadal (periovaric, epididymal), RP/R = retroperitoneal. Statistical significance of the differences between groups (2-way anova): the columns show the P values for sex, diet and their interaction. A superscript ^f^ represents higher (overall) values for females and ^m^ for males. The arrows show the direction of significant changes for diet: ↓ indicate (overall) slower values for cafeteria diet-fed rats; a i in the interaction column indicates a significant interaction between the two factors analysed. The statistical significance of differences between sites was estimated by a 3-way-ANOVA; post-hoc Duncan test (same conventions as in Table 1)

The expressions of the enzymes controlling the critical step of conversion of 3C pyruvate to 2C acetyl-CoA, *Pdk4* and *Pdk2*, showed little change because of sex, with only higher male values for SC in *Pdk4* and PG in *Pdk2*, and no changes at all induced by the cafeteria diet (with only a significant decrease in ME for *Pdk2*).

The expressions of the main genes controlling enzymes of lipid metabolism are presented in Table 6. The effect of "site" was significant for all genes studied with the exception of Acly, which was not. The differences related to sex were not evenly distributed, with higher expression values for males, the exceptions being Acc1 (ME and RP) and *Acly* (ME) which showed higher specific mRNA concentrations in females. In all other cases, males showed higher mean values: *Fas* (SC and RP), *Lpl* (SC), *Atgl* (SC and PG), *Cpt1* (all sites, except ME), *Cpt2* (PG) and *Lcad* (SC).

**Table 6:** Gene expression in WAT of control and cafeteria diet-fed rats in fmol/g protein. II Lipid metabolism.

protein	gene	WAT	male control	male cafeteria	female control	female cafeteria	P site	P sex	i	P diet
acetyl-CoA carboxylase 1 EC 6.4.1.2	*Acc1*	SC	74.0±16.3	59±6	149±37	40.3±5.0	0.0020R>S≈M≈P	NS		0.0024↓
		ME	30.6±4.0	14.6±1.6	165±49	9.2±2.1		0.0134^f^	i	0.0019↓
		PG	126±12	53.4±9.5	156±66	28.0±4.6		NS		0.0064↓
		RP	81.0±7.2	33.5±4.8	542±206	27.6±2.7		0.0087^f^	i	0.0019↓
fatty acid synthase EC 2.3.1.85	*Fas*	SC	7620±4110	8762±1955	2447±403	540±47	<0.0001 S>M≈P≈R	0.0202^m^		NS
		ME	192±74	110±22	1660±536	67.3±13.9		NS	i	0.0268↓
		PG	1269±250	701±166	2106±795	319±45		NS		0.0056↓
		RP	572±85	204±28	3318±1130	190±32		0.0050^m^	i	0.0007↓
ATP: citrate lyase EC 4.1.3.8	*Acly*	SC	167±81	174±43	186±54	71.5±7.7	NSS>M	NS		NS
		ME	22.2±2.9	9.9±2.3	160±34	6.4±1.5		0.0020^f^	i	0.0003↓
		PG	109±10	88.6±13.9	228±102	39.9±6.3		NS		0.0391↓
		RP	25.1±4.7	14.6±2.4	295±132	13.3±2.6		NS		NS
hormone-sensitive lipase EC 3.1.1.79	*Hsl*	SC	631±131	648±138	486±37	660±63	<0.0001 S≈P>R>M	NS		NS
		ME	166±38	130±23	218±61	110±12		NS		NS
		PG	674±59	670±70	664±64	461±45		NS		NS
		RP	458±39	344±49	645±84	295±34		NS	i	0.0002↓
lipoprotein lipase EC 3.1.1.34	*Lpl*	SC	6300±1305	6700±1110	3790±440	4520±670	<0.0001 S>P>M≈R	0.0365^m^		NS
		ME	1190±38	1630±470	1100±80	1430±260		NS		NS
		PG	3970±720	3790±440	4320±290	2080±250		NS	i	0.0209↓
		RP	2610±350	1750±110	3440±820	1470±150		NS		0.0052↓
triacylglycerol lipase (adipose type) EC 3.1.1.3	*Atgl*	SC	1320±300	1680±270	701±26	1470±150	<0.0001 S,P>R>M	0.0426^m^		NS
		ME	429±95	412±92	338±95	403±79		NS		NS
		PG	1450±300	1790±290	1040±150	1180±90		0.0332^m^		NS
		RP	700±48	608±74	732±51	536±68		NS		0.0400↓
carnitine palmitoyl-transferase (liver type) EC 2.3.1.21	*Cpt1*	SC	39.6±14.8	58.9±9.0	19.4±3.9	37.9±7.3	<0.0001 P>S>M>R	0.0426^m^		NS
		ME	14.0±2.7	3.8±0.9	13.7±3.6	0.7±0.1		NS		0.0001↓
		PG	16.3±1.7	18.9±1.1	10.8±1.7	7.7±1.2		<0.0001^m^		NS
		RP	3.3±0.5	3.4±0.5	2.0±0.4	2.6±0.2		0.0200^m^		NS
carnitine palmitoyl-transferase (muscle type) EC 2.3.1.21	*Cpt2*	SC	10.6±3.0	17.9±2.8	17.0±1.8	13.4±12.7	<0.0001 P>S>M>R	NS	i	NS
		ME	10.7±1.5	7.8±1.0	14.9±3.6	7.5±1.3		NS		0.0214↓
		PG	25.1±4.3	20.5±1.7	19.2±3.4	11.7±1.7		0.0332^m^		NS
		RP	13.4±1.7	11.3±1.7	20.9±3.4	10.5±1.9		NS		0.0130↓
long-chain acyl-CoA dehydrogenase EC 1.3.8.8	*Lcad*	SC	1076±347	1370±155	365±23	733±117	<0.0001 S>P>M≈R	0.0037^m^		NS
		ME	166±9	196±14	298±60	179±32		NS		NS
		PG	353±72	653±99	412±91	359±37		NS		NS
		RP	204±22	379±29	302±49	207±29		NS		NS

The data are presented as fmol of the corresponding mRNA for g of tissue protein, and are the mean ± sem of 6 different animals. Up to three significant digits are shown for each mean value. SC/S = subcutaneous inguinal: ME/M = mesenteric; PG/P = perigonadal (periovaric, epididymal), RP/R = retroperitoneal. Statistical significance of the differences between groups (2-way anova): the columns show the P values for sex, diet and their interaction. A superscript ^f^ represents higher (overall) values for females and ^m^ for males. The arrows show the direction of significant changes for diet: ↓ indicate (overall) slower values for cafeteria diet-fed rats; a i in the interaction column indicates a significant interaction between the two factors analysed. The statistical significance of differences between sites was estimated by a 3-way-ANOVA; post-hoc Duncan test (same conventions as in Table 3). P values >0.05 are presented as NS.

The effects of diet resulted in all cases in either no change or decreased expression; no increased expressions were observed when comparing cafeteria diet and controls. In the case of *Acc1*, all sites showed significant effects of diet, and in *Fas*, all showed significant changes except SC. *Acly* decreased its expression with diet in ME and PG; *Hsl* showed changes only in RP, *Lpl* in PG and RP. *Atgl* also decreased its expression in RP; *Cpt1* in ME and *Cpt2* in RP and ME; there were no changes at all in *Lcad*.

**Fig 5:**
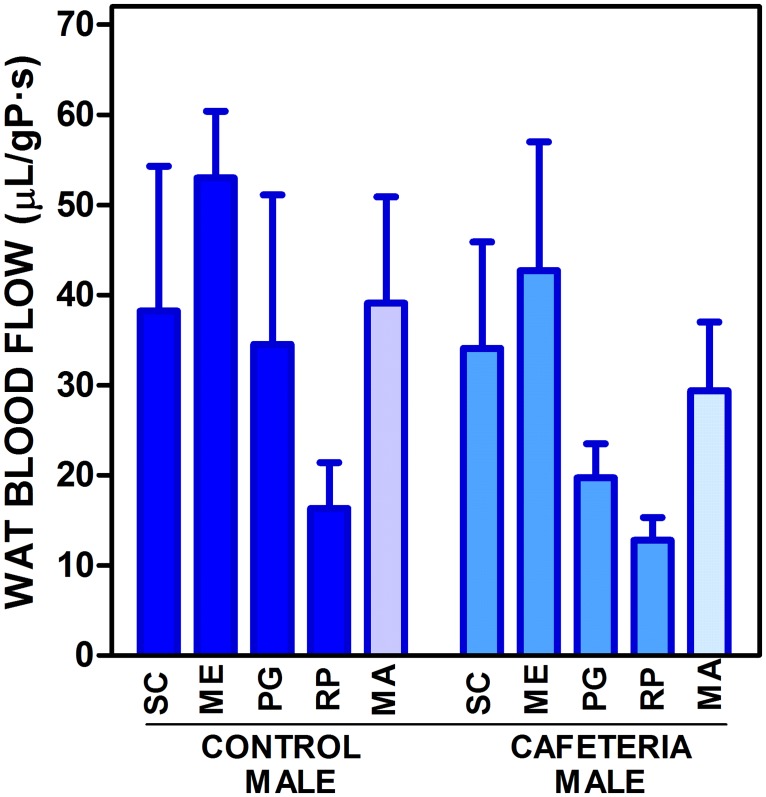
WAT blood flow in male rats fed control or cafeteria diets. The data are the mean ± sem of 6 animals per group, and are expressed in μL/s·gP (g of tissue protein). The conventions used are the same as in Fig. 1; MA = mean value for SC, MS, PG and RP taking into account their combined masses.

The estimation of WAT blood flow in male rats fed the control or cafeteria diets is shown in Fig. 5. The data are referred to g of tissue protein for direct comparisons with the other Figures and Tables. There was a trend to show lower blood flows for the WAT sites, including the composite value of all four, MA, in cafeteria diet-fed rats but there were no statistically significant differences either for the four studied sites or their composite; there was no significant difference for "site" either.

Table 5 also presented the expression of the most abundant gene for an isoenzyme of nitric oxide synthase (endothelial type) *Nos3*. Sex showed significantly higher values for males in SC and PG. Feeding a cafeteria diet increased the expression of *Nos3* in SC, and decreased it in ME and RP.

## Discussion

The production of lactate from glucose by WAT is well known [26–28], and is related to glucose availability [46] in a way similar to that of the Cori cycle [5] between muscle and the splanchnic bed. However, there are reports on the active use of lactate as lipogenic substrate by WAT [6,7]. The release of lactate from human and rat subcutaneous WAT has been proven *in vivo* [26,47], and we have observed, in cultured 3T3L1 adipocytes [25], that these cells produced an inordinately large amount of lactate when medium glucose concentration was high. A number of elegant studies, have found that the oxygen consumption by WAT under basal conditions is low, justifying a normal operation of the tissue even at low concentrations of oxygen [29,30,48].

The 3T3L1 adipocytes converted almost quantitatively glucose to lactate, which represents a negligible consumption of oxygen under conditions of normoxia [25]. Consequently, the hypothesis that limited blood (and thence oxygen) supply to WAT, a mechanism of defence against excess energy substrate availability [49], may elicit inflammation because of hypoxia [50,51] should be revised, at least for adipocytes, the main component of WAT.

In this study, we found a considerable difference in the potential for lactate production between different WAT sites as shown by different levels of activity and gene expression for lactate dehydrogenase. A high enzyme activity is no proof in itself that lactate is being produced in a given tissue without using dynamic (albeit invasive) tracer studies. However, the data presented in Fig. 4 show that there is a close correlation between WAT lactate content and lactate dehydrogenase activity, regardless of site, sex and dietary treatment. These data strongly suggest that the actual lactate tissue levels are a consequence of the lactate dehydrogenase activity.

Furthermore, the plasma/tissue molal lactate ratios proved that, in all samples, lactate concentration was higher in water tissue than in plasma, i.e. the lactate in WAT could not come from plasma (uphill gradient) but was produced in WAT and released to plasma. Evidently, the main ultimate destination of this lactate is the liver, where it may be used for gluconeogenesis, lipogenesis or oxidized to supply energy. Gluconeogenesis is highly improbable under conditions of excess glucose [52]. Oxidation to CO_2_ for energy is improbable too in liver and other tissues because of the availability of glucose and the induction of insulin resistance by lactate itself [53]. The large presence of fatty acids in cafeteria rats further increased insulin resistance [54]. In consequence, the most probable fate for most of the lactate produced by adipocytes will be its incorporation to the hepatic lipogenic pathway, and their final release as lipoprotein triacylglycerols.

Adipose tissue is one of the main body producers of lactate [13]. In postabsorptive state humans, whole adipose tissue releases 60–150 μmol/min; other important lactate producing tissues are the brain (with a contribution of around 50 μmol/min) and skeletal muscle. However, although skeletal muscle is a main site of lactate production during exercise [5], it also plays an important role in lactate clearance under basal conditions [13].

It is unclear why WAT contains both muscle and heart type lactate dehydrogenase isoenzymes, (HHMM) with different affinities for lactate and markedly distinct functions in different organs [55]. In the present conditions, both *Ldha* and *Ldhb* genes behaved in a similar way and both (and especially their combined expression) was closely adjusted to the total enzyme activity measured, which points to a similar (or shared) regulation *in vivo*, and also to a gene expression-linked mechanism of regulation of the enzyme activity. The similar turnover rate of the isoenzymes [55] agrees with this interpretation.

The limited changes in circulating lactate suggest that its turnover in blood was fast [56] in agreement with the high capacity of liver to take up and metabolize lactate [57] as indicated above. However, given the relatively large mass of adipose tissue, at least in the overweight cafeteria diet-fed rats, the effect of a significant breakup of glucose to produce lactate is far from being negligible and undoubtedly may help downregulate glycaemia in a significant proportion, at least under basal, i.e. non-exercise, conditions.

Intake of a hyperlipidic cafeteria diet results in a proinflammatory state [58], which is often associated with hypoxia [59,60]. However, contrary to what was expected, no increases in lactate dehydrogenase activity, neither of tissue lactate concentrations were found in the WAT of cafeteria diet-fed rats. The close correlation observed between lactate dehydrogenase activity and tissue lactate content, irrespective of site, sex and diet, suggests that hypoxia could not be a critical factor in the regulation of lactate production. This is again in agreement with the need to revise the purported relationship between hypoxia and inflammation.

There were sex-related differences in the expression of a number of key enzymes related to lipid synthesis, but the most marked effects were attributable to the exposure to cafeteria diet, providing an excess of energy. The consequence was an increased WAT mass [61], via increases in both cell numbers and cell size, as observed here and in agreement with previous studies [62]. However, the factor "site" was markedly relevant for practically all parameters studied. The data on the expression of genes involved in lipogenesis from glucose show clearly that cafeteria diet had a marked overall influence; the entry of glucose into WAT cells was probably limited, as shown by lower *Glut4* and *Hk2* expressions. This decrease may be part of the tissue mechanism of defence against a sustained excess of energy supply and the generalized insulin resistance [63] that makes WAT the ultimate destination for excess circulating glucose [49,64]. The relative lack of changes in the expression of pyruvate dehydrogenase kinases suggest that the oxidation of pyruvate to acetyl-CoA was practically unaffected by diet, i.e. this was not, probably, an outlet for excess glucose carbon towards lipogenesis, at least in WAT. Notwithstanding, the maintenance of these expressions unchanged also hinted to a limited success of glucose transporter 4 and hexokinase decreases in gene expression to stem the flow of glucose into the cells. All the genes controlling lipogenesis, from *G6pd* role in the production of NADPH, to the cytoplasm supplier of acetate *Acly*, and to the proper lipogenic enzyme genes *Acc1*, and *Fas*, showed steady decreases in expression under the hyperlipidic cafeteria diet, as previously observed [22,65]. Lipoprotein lipase expression was also decreased, but only in RP and PG. The limitations induced in lipid metabolism by cafeteria feeding were less marked on the lipase genes *Hsl* and *Atgl*, which showed decreases only in RP.

WAT lipid utilization, thus, was also limited under cafeteria feeding, an obvious conclusion because of increases in the rat global fat mass and in all WAT sites. The expression of genes related to the transfer of acyl-CoA to the mitochondria was also limited (*Cpt1* and *Cpt2*), and those controlling the oxidation of fatty acids was unchanged (albeit maintained at low levels) as shown by the lack of effect of diet on *Lcad* expression. These data agree with those of cultured adipocytes, in which the use of glucose as substrate for lipogenesis was largely shunted to the production of 3C (lactate) fragments [25] for eventual exportation to the liver. However, the increase in WAT mass and triacylglycerol accumulation of cafeteria rats is difficult to explain with lowered WAT glucose uptake, limited lipogenesis and maintained lactate production capability, unless, incorporation of blood-borne lipids is factored in.

At first, we assumed that the contrast between lactate levels and lactate dehydrogenase activity in a given WAT site could be due to differences in blood flow. The control of blood flow is probably a key mechanism of control of substrate access to WAT [49,66,67], and its limitation under conditions of excess available energy is counterbalanced by the eventual hypoxic effects induced by this limitation [66,68]. The higher production of NOx in the obese [69] and its effects countering the vasoconstriction elicited by other factors [70,71] agrees with that interpretation. We have observed decreases in WAT expression of *Nos3*, the endothelial nitric oxide synthase gene [72], a key controller of blood flow [73], in most sites of rats fed the cafeteria diet; but it increased in subcutaneous WAT. However, the blood flow study (done only in males to limit animal lives and costs), showed a limited variability (not significant) from site to site in this parameter, which hints to a general uniform control of WAT blood flow comparable to the ability to uniformly depose fat in all WAT sites and other organs [22] under comparable energy availability conditions.

In any case, the data on blood flow did not explain the differences in lactate and lactate dehydrogenase activity. In spite of the efforts invested, no proof has been found linking blood flow (or its control) to WAT lactate production or release. Thus, lactate production by WAT seems to be essentially unrelated to hypoxia and inflammation.

A main conclusion of this study is the production of lactate by WAT irrespective of site, diet or sex, and that this production is a direct consequence of lactate dehydrogenase activity in the tissue. Furthermore, this activity is a direct correlate of the main lactate dehydrogenase controlling genes' expression. In sum, the ability to produce lactate by WAT is not directly dependent of WAT metabolic condition. We postulate that a main function of the lactate dehydrogenase path of WAT may be that of converting excess circulating glucose to 3C fragments as a way to control glycaemia and/or providing shorter chain substrates for use as energy source elsewhere. The higher circulating lactate levels of obese humans [27] supports this interpretation, and suggests a potential role of WAT in the control of glycaemia, at least in obese individuals.

More information must be gathered before a conclusive role of WAT in the control of glycaemia and the full existence of a renewed glucose-lactate-fatty acid cycle [74] is definitely established.
